# Resurgence of an international hepatitis A outbreak linked to imported frozen strawberries, Germany, 2018 to 2020

**DOI:** 10.2807/1560-7917.ES.2020.25.37.1900670

**Published:** 2020-09-17

**Authors:** Claudia Ruscher, Mirko Faber, Dirk Werber, Klaus Stark, Julia Bitzegeio, Kai Michaelis, Daniel Sagebiel, Jürgen J Wenzel, Julia Enkelmann

**Affiliations:** 1 State Office for Health and Social Affairs (LAGeSo), Berlin, Germany; 2 Robert Koch Institute, Department for Infectious Disease Epidemiology, Berlin, Germany; 3 National Consultant Laboratory for Hepatitis A Virus and Hepatitis E Virus, Institute of Clinical Microbiology and Hygiene, University Medical Center Regensburg, Regensburg, Germany

**Keywords:** Germany, disease outbreaks, Europe, foodborne diseases, genotype, Hepatitis A virus, sequence analysis

## Abstract

Following outbreaks linked to frozen strawberries in Sweden and Austria in 2018, 65 cases linked to the same hepatitis A virus strain were detected in Germany between October 2018 and January 2020, presenting in two waves. Two case–control studies and a comparison of cases’ consumption frequencies with purchase data from a large consumer panel provided strong evidence for frozen strawberry cake as the main vehicle of transmission. Of 46 cases interviewed, 27 reported consuming frozen strawberry cake and 25 of these identified cake(s) from brand A spontaneously or in product picture-assisted recall. Trace back investigations revealed that the Polish producer involved in the previous outbreaks in Sweden and Austria had received frozen strawberries from Egypt via a wholesaler that also delivered frozen strawberries to manufacturer of brand A. Phylogenetic analyses linked the outbreak strain to similar strains formerly isolated from sewage, stool and strawberries in Egypt. Complete trace back and timely recall of products with strong evidence of contamination is important to control an outbreak and prevent later resurgence, particularly for food items with a long shelf life. Continued molecular surveillance of hepatitis A is needed to identify outbreaks and monitor the success of food safety interventions.

## Background

Hepatitis A virus (HAV) causes acute inflammatory hepatic infections in humans. Transmission occurs primarily via the faecal-oral route through contaminated food or water or person-to-person spread [1]. While the disease is often asymptomatic or mild in younger children, it can cause debilitating symptoms and fulminant hepatitis in adults. After an abrupt onset with fever, malaise and abdominal discomfort, jaundice is the predominant symptom. The average incubation period is 28 to 30 days (range: 15–50) with maximum infectivity during the latter half of the incubation period, i.e. while being asymptomatic [1]. HAV retains infectivity after freezing and can persist in the environment, being able to withstand food-production processes routinely used to inactivate bacterial pathogens [2].

Direct or indirect detection of HAV infection in humans is notifiable in Germany to local public health authorities (LPHA), which transmit case reports electronically via the state level to the national public health institute (Robert Koch Institute, RKI). The case definition of hepatitis A in place for surveillance purposes in Germany is: a symptomatic disease (defined as one or more of the following: fever, abdominal discomfort, increase of serum transaminases, jaundice), plus laboratory confirmation (i.e. detection of HAV nucleic acid or HAV-specific IgM or a distinct increase of HAV-IgG concentrations). Symptomatic cases with an epidemiological link to a laboratory-confirmed hepatitis A case also fulfil the case definition. Forwarding of HAV-reactive serum or stool samples from diagnosing laboratories to the National Consultant Laboratory for HAV for sequencing is voluntary, and is intensified during outbreaks.

In 2016, the European Union/European Economic Area (EU/EEA) incidence of hepatitis A was 2.4 cases per 100,000 population; the highest incidences were in eastern EU countries, while HAV infections in other EU/EAA regions were mostly associated with infections acquired abroad [3]. Like most high-income countries, Germany is a low-incidence country with a median hepatitis A incidence of 1.02 per 100,000 inhabitants between 2010 and 2018. The median hepatitis A incidence in Berlin, Germany is slightly higher (1.79/100,000 in 2010–2018). European outbreaks of hepatitis A among men who have sex with men caused higher case numbers in Berlin and the EU/EEA in 2017 [4,5]. Recently, several hepatitis A outbreaks associated with frozen berries have been described in Europe [6,7].

## Outbreak detection

 This HAV subgenotype IB strain has previously caused outbreaks in Sweden (June–July 2018) and Austria (July–September 2018) [8], comprising a total of 34 reported cases. Combined epidemiological and microbiological outbreak investigations identified imported frozen strawberries produced in Poland as the vehicle. In Sweden, the HAV outbreak strain was detected in frozen strawberries and the contaminated batch was withdrawn from the Swedish market. Trace-back investigations from both Sweden and Austria identified Polish producer Y as the source for the implicated frozen strawberries.

Shortly after outbreak control in both countries, cases with the identical virus sequence started to appear in Germany in October 2018. The Federal Office of Consumer Protection and Food Safety (BVL) and all federal public health authorities were informed about this by the RKI and sequencing of samples of autochthonous hepatitis A cases was intensified.

Here we describe the results of the epidemiological investigation of an outbreak of hepatitis A in Germany presenting in two waves, one in 2018 and another in 2019.

## Methods

### Outbreak case definition

A confirmed outbreak case was defined as a person with a HAV infection fulfilling the surveillance case definition, notified to German local public health authorities (LPHAs) from September 2018 to 28 February 2020, and with a sequence identity ≥ 99.4% to the outbreak strain (EPIS UI-487, GenBank: MH730560), based on a 460-nt fragment of the VP1/P2A region as suggested by the HAVnet unified typing protocol (≤ 2 mismatches in 460 nt). Probable cases were those epidemiologically linked to a confirmed case, but were not sequence-confirmed. Cases with disease onset more than 2 weeks after disease onset of a hepatitis A case belonging to the outbreak in the same household were considered secondary.

### Enhanced surveillance and case finding

Cases were identified by the mandatory notification system combined with genotyping results of the National Consultant Laboratory for HAV in Regensburg. LPHAs were asked to request the forwarding of serum samples of cases from notifying primary laboratories to the National Consultant Laboratory for HAV.

A first wave of cases started in 2018. Cases were asked to complete a questionnaire (interview or self-completed) on purchasing behaviour and consumption of food products containing berries, with focus on frozen strawberries in the 2 months before illness. If such an item was consumed, cases were asked to identify place of purchase and specific products. Product pictures were provided to assist recall. Except for a single case with disease onset in April 2019, no further outbreak cases with disease onset in the first half of 2019 were detected, despite enhanced molecular surveillance of HAV.

A second wave of cases began in July 2019, when HAV notification rates in Berlin increased from a median of one case per week to 12 cases in one week, i.e. between 1 and 7 July. Berlin LPHAs immediately requested laboratories to store diagnostic specimens of those and future cases, and to forward them to the National Consultant Laboratory for HAV for molecular confirmation and sequence typing. On 8 July, the State Office for Health and Social Affairs Berlin (SOHSA) and LPHAs started hypothesis-generating telephone interviews with cases to identify potential sources of infection using a questionnaire designed for the outbreak investigation. The questionnaire focused on food consumption known to be associated with HAV infection, e.g. fresh fruit and vegetables, fish and seafood, frozen fruit and frozen berry products, and restaurant visits 2 months before disease onset. Interviews started before sequencing results were available.

### International communication

Information about the outbreak was communicated since June 2018 via the European Centre for Disease Prevention and Control (ECDC)’s Epidemic Intelligence Information System (EPIS) for food- and waterborne diseases and zoonoses.

### Case–control studies

Two independent case–control studies (CCS) were conducted to investigate associations of different food items containing frozen strawberries and hepatitis A; one in each wave.

The first CCS (CCS1) was conducted by the RKI and included all 21 primary outbreak cases identified in Germany with disease onset in 2018 (i.e. during the first wave) that provided information on food consumption 2 months before symptom onset. Participants reported disease onset between 5 September and 22 December 2018. To obtain rapid estimates of background exposures, convenience controls among employees of the RKI were recruited. On 17 December 2018, all 237 employees of the Department of Infectious Disease Epidemiology included in the mailing list were asked to complete an emailed questionnaire on consumption of products containing frozen strawberries within the same time period as cases.

The second case–control study (CCS2) was conducted by the SOHSA and included the first 11 outbreak cases of the second wave in Berlin with disease onset between 13 June and 26 June 2019. Employees of two divisions of the SOHSA were invited as controls (n = 103). They were asked to answer an online-survey on consumption of products containing frozen strawberries 2 months before the median date of symptom onset of the cases.

The association between hepatitis A and consumption of food items was estimated by univariable logistic regression calculating odds ratios (OR) and 95% confidence intervals (CI). Associations were considered statistically significant if p ≤ 0.05. Stata version 15 was used to analyse data (StataCorp, Texas, United States).

### Consumer panel data for estimation of background exposure

We compared consumption frequencies for food items of cases identified in the case–control studies with purchase data from a large consumer panel. The probability of finding at least as many persons who ate the suspected item as cases reported in the CCS was estimated using a standard binomial model. To obtain estimates of the exposure prevalence, i.e. the background probability, we acquired data from a large consumer panel (n > 20,000) in Germany, operated by GfK (https://www.gfk.com). Participants of this panel are responsible for grocery shopping in the household, scan each food item they purchase and send the scan to GfK every day where data are collated and weighted to be representative for each federal state and for Germany as a whole. To best resemble the likely exposure period of our cases, we chose a 2-month interval that we derived by subtracting the average incubation period from the median date of symptom onset in both case series and added as well as subtracted one month from that date (resulting in 18 August 2018–18 October 2018 for CCS1 and 26 April 2019–26 June 2019 for CCS2). We obtained data from panel persons aged between 20 and 74 years (similar to the age range of cases). We chose the entire panel for CCS1, and geographically restricted the panel to participants living in Berlin for CCS2.

### Microbiological investigations

In the National Consultant Laboratory for HAV, quantitative reverse-transcription PCR (lower limit of detection 837 IU/ml) was performed with primers and probe as published by Costafreda et al. [9]. Molecular typing of the VP1/P2A genomic region was performed according to the unified HAVNET protocol (http://www.rivm.nl/en/Topics/H/HAVNET) as described previously [10]. The European Nt Archive (ENA) database was searched for sequences similar to the HAV outbreak strain using the FASTA algorithm. A rooted maximum likelihood phylogenetic consensus tree for VP1/P2A sequences was inferred using Randomized Axelerated Maximum Likelihood (RAxML) version 8.2.11 (http://sco.h-its.org/exelixis/web/software/raxml).

### Trace back and food investigation

German food safety authorities were regularly informed about the updated results of the epidemiological investigations.

Information communicated via the EU’s Rapid Alert System for Food and Feed (RASSF) platform concerning this outbreak is included in this report.

### Ethical statement

The CCS were conducted within the framework of the German Infection Protection Act (IfSG) as part of an outbreak response and public health practice. Mandatory regulations were respected, and thus review by an ethics committee was not required.

## Results

**Figure 1:**
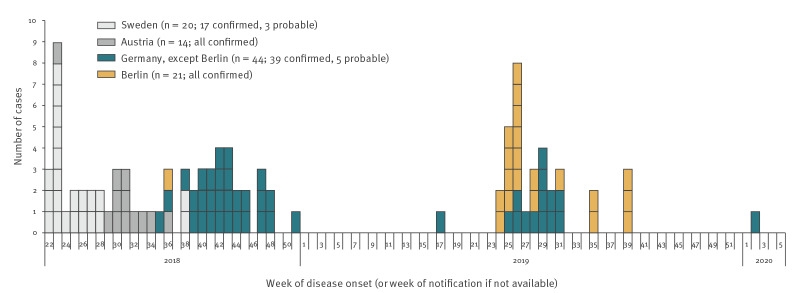
Hepatitis A outbreak cases by week of symptom onset, Sweden^a^, Austria^a^ and Germany, 2018–2020

^a^ Data extracted from Enkirch et al. [8].

As of 28 February 2020, 65 cases belonging to the outbreak had been reported in Germany (Figure 1). The median age was 48 years (range: 1–77) and 45% were female. Most patients (77%) were hospitalised (Table 1). Interviews were conducted with 46 cases, of which 34 reported definite and four reported possible consumption of products containing frozen strawberries. Frozen strawberry cake was the most commonly mentioned strawberry product; 27 cases reported definite and five reported possible consumption. Of the 27 cases that reported definite consumption of frozen strawberry cake, 26 provided details on the type of cake, with 25 of these identifying strawberry cake(s) from brand A spontaneously or in product picture-assisted recall.

**Table 1:** Characteristics of confirmed and probable hepatitis A outbreak cases, Germany, 2018–2020 (n = 65)

Characteristics	First wave (disease onset August–December 2018) (n = 30)	Second wave (disease onset June–September 2019) (n = 33)	Outliers^a^ (n = 2)	Total (n = 65)
Interviewed^b^	21	23	2	46
Female	15	13	1	29
Male	15	20	1	36
Median age, years (range)	48 (9–73)	52 (1–77)	NA	48 (1–77)
Hospitalised	24	24	2	50

NA: not applicable.

^a^ One case with disease onset in April 2019 and one with disease onset in January 2020.

^b^ Primary cases.

### First wave

Overall, 30 cases (27 confirmed and three probable) from 11 states had a disease onset between 29 August 2018 and 22 December 2018. Of the confirmed cases, three were likely secondary infections.

Despite enhanced molecular surveillance, no further outbreak cases with disease onset in 2019 were detected, except for a single case in April, until June 2019, when the outbreak strain resurged, this time predominantly affecting Berlin.

### Second wave

During the second wave, 33 cases (31 confirmed and two probable) from seven states had disease onset between 13 June 2019 and 29 September 2019. One probable and one confirmed case probably represented secondary infections. The majority of cases were notified in Berlin (n =20) and the neighbouring state Brandenburg (n = 5). Eight cases were notified from five other states in different parts of Germany. One case reported travel to Berlin.

In Berlin, 25 of 38 notified hepatitis A cases between July and October 2019 underwent sequence analysis. Of these, 20 belonged to the outbreak.

### Post second wave

Post abatement of the second wave, a single case with disease onset in January 2020 was identified. The case reported very likely having consumed a frozen strawberry cake of brand A in the incubation period. The cake had been purchased in summer 2019.

### International communication

An urgent inquiry on EPIS was posted in June 2018 by Sweden. Germany reported its first cases with the outbreak sequence in October 2018. Since then, the Netherlands reported two cases with identical sequence: one with disease onset in September 2018 after travel to Germany and one in May 2019. Both had consumed strawberries (no information if fresh or frozen). More recently, Italy has also reported two cases with identical sequence in August and September 2019; both had consumed frozen berries.

### Case–control studies

In CCS1, 78 controls participated (33% response rate), 82% (n = 64) were female. The median age of the 21 primary outbreak cases (10 male and 11 female) that were included in CCS1 was 52 years (range: 9–73). Univariable logistic regression showed that cases more frequently reported consumption of frozen strawberry cake (OR: 43; 95% CI: 11–171), especially from brand A (OR: 32; 95% CI: 8.0–125) than controls. Compared with controls, cases also more frequently reported consumption of frozen strawberries and strawberry smoothies (Table 2). Of the 21 cases, 14 reported definitely and one reported possibly having eaten frozen strawberry cake in the 2 months before illness; 13 (12 definite and the one with possible consumption) from one particular brand (brand A). Of those 13, nine cases identified at least one specific cake; most commonly cake X (n = 6), cake Y (n = 4) and cake Z (n = 3). All of these cakes are ready to eat after thawing and do not require oven cooking. Of the six cases that did not report definite consumption of strawberry cake, four had eaten at least one other frozen strawberry product (strawberry smoothies (n = 3), frozen strawberries (n = 2)).

**Table 2:** Association between hepatitis A and food consumption based on univariable logistic regression in two case–control studies, Germany, 2019

Exposure	Cases			Controls			OR	95% CI	p value
	Total	Exposed (n)	Exposed (%)	Total	Exposed (n)	Exposed (%)			
**Case**–**control study 1 (cases = 21; controls = 78)**									
Frozen strawberry cake^a^	20	14	70	77	4	5	42.6	10.6–170.7	< 0.0001
Frozen strawberry cake from brand A^a^	19	12	63	78	4	5	31.7	8.0–125.0	< 0.0001
Frozen strawberries^a^	16	6	38	71	10	14	3.7	1.1–12.3	0.036
Strawberry smoothies^a^	14	5	36	74	11	15	3.2	0.9–11.3	0.073
**Case–control study 2 (cases = 11; controls = 33)**									
Frozen cake	11	8	73	28	4	14	15.3	2.8–83.9	0.002
Frozen strawberry cake^b^	11	5	45	28	3	11	6.4	1.2–34.6	0.031
Red fruit jelly	11	4	36	32	3	9	5.5	0.9–30.5	0.050
Frozen berries (containing strawberries)	11	3	27	26	6	23	1.3	0.2–6.3	0.786

CI: confidence interval; OR: odds ratio.

^a^ Possible consumption was coded as missing for this analysis.

^b^ In picture-assisted interviews after conducting the case–control study, all eight cases who reported consumption of frozen cake identified frozen strawberry cake X from brand A.

In CCS2, 33 controls responded (32% response rate). Demographic information on sex and age was not available for controls in CCS2. The median age of the first 11 outbreak cases of the second wave was 38 years (range: 5–74) with a balanced sex ratio (six female, five male). Univariable logistic regression showed that cases more frequently reported consumption of frozen cake (8/11 cases; OR: 15; 95% CI: 3–84) and frozen cake with strawberries (5/11 cases; OR: 6; 95% CI: 1–35) than controls (Table 2).

After conducting CCS2, follow-up interviews with 15 of 18 Berlin outbreak cases using product pictures revealed that 10 cases reported consumption of frozen cakes, all of them frozen strawberry cake X from brand A. Six had exclusively eaten cake X, and four had eaten cake X and other cakes of brand A. An additional case had only consumed frozen strawberries.

### Consumer panel data for estimation of background exposure

In the panel, 2.2% (772/34,838) (CCS1) and 3.8% (64/1,696) (CCS2) of participants respectively, bought frozen strawberry cake from brand A during the specified 2-month periods chosen for the CCS.

Thus, the probability of finding at least 12 of 19 persons in CCS1 and 8 of 11 in CCS2 chosen at random to have eaten frozen strawberry cake from brand A is less than one in a billion (10^-9^), assuming that 2.2% (CCS1) and 3.8% (CCS2) of people (purchased and) ate that product in a 2-month interval. Even when assuming a background probability to be 40% to account for various possible biases, e.g. residual confounding because of insufficiently accounting for age, or underascertainment because of incomplete product scanning and the fact that the product could have been eaten outside the household, the probability would still be < 0.05.

### Microbiological investigations

**Figure 2:**
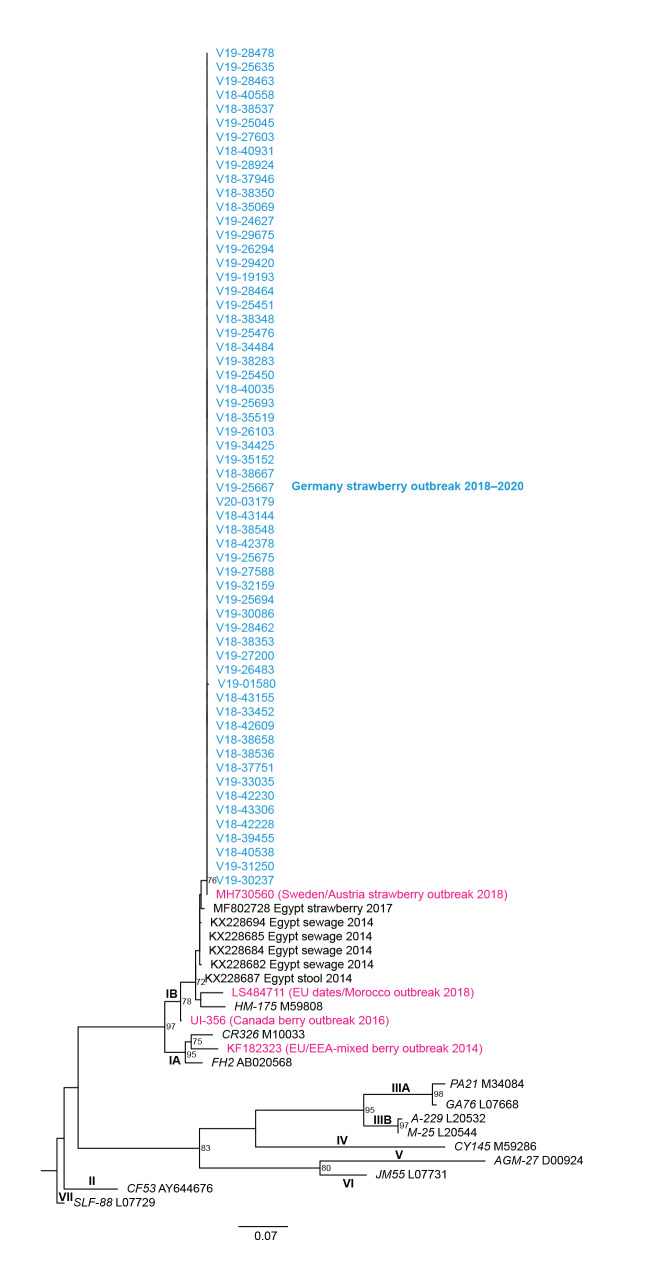
Phylogenetic tree of 60 hepatitis A virus sequences from outbreak cases, Germany, September 2018–January 2020

The sequences from 58 cases clustered with the recent HAV subgenotype IB outbreak strain JT9763SWE2018 from Sweden/Austria (100% similarity, MH730560, Figure 2). The next highest similarities referred to specimens from Egypt: strawberries from 2017 (98.7%, 6-nt mismatch over 460-nt, MF802728), as well as samples from wastewater (sewage from 2014: 98.6–98.9%, KX228682, KX228684, KX228685 and KX228694 and stool from 2014 (98.6%, KX228687) [11,12].

The outbreak sequences cluster in hepatitis A virus (HAV) subgenotype IB and are 100% similar to sequence MH730560 from Sweden/Austria. Outbreak cases, which are highlighted in blue, were analysed by molecular typing of 460 nt of the VP1/P2A region. The six most closely related European Nt Archive (ENA) database entries were from Egyptian strawberries, stool and sewage. Typical reference members of genotype I–VII are denoted by isolate name (italics) and database ID. Genotype VII was used as an outgroup. Numbers at the nodes indicate bootstrap values of > 60%. The Sweden/Austria outbreak sequence and three additional sequences from selected food associated hepatitis A outbreaks are shown in magenta. The scale bar represents 0.07 substitutions per site.

### Trace back and food investigation

Enkirch et al. reported that trace back investigations in both Swedish and Austrian outbreaks led to the same Polish producer Y [8]. It was communicated via the RASSF that the batch that tested positive in Sweden was only sold to Swedish recipients and contained only strawberries originating from Poland. No deliveries from producer Y to Germany could be identified, and both Sweden and Austria reported they had not delivered implicated frozen strawberries to Germany.

**Figure 3:**
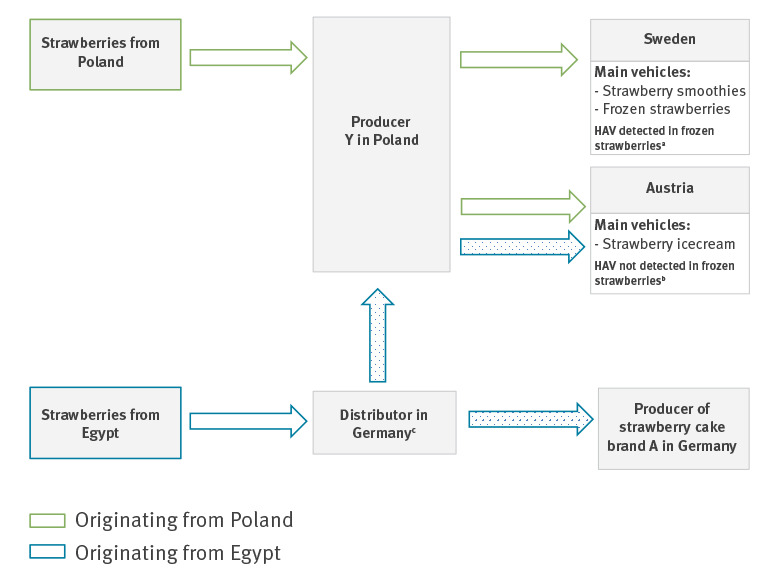
Trade relationships regarding frozen strawberries according to Enkirch et al. [8] and RASFF 2018.1813, 2018

However, during expanded trace back investigation after outbreak resurgence in Germany, shipments of frozen Egyptian strawberries to producer Y by a German distributor were identified. According to RASFF 2018.1813 [13], the investigation revealed that producer Y distributed these strawberries to Austria in April 2018 and the German distributor dispatched frozen strawberries of the same batch within Germany where they entered a production of frozen strawberry quarters for the manufacturer of cake brand A. A retention sample of frozen strawberries of that batch provided by the German distributor tested negative for HAV (Figure 3).

RASFF: Rapid Alert System for Food and Feed.

^a^ In leftover strawberries, the hepatitis A virus (HAV) IB outbreak strain was detected. The strain is phylogenetically related to strains from Egypt.

^b^ Tested strawberries originated from Poland.

^c^ A retention sample of frozen strawberries was tested for HAV in summer 2019 and no HAV was detected.

No claim to be exhaustive. The figure is based on information available via RASFF and Enkirch et al. [8]; additional, unknown or unpublished trade relationships are not displayed.

Dotted arrows indicate strawberries coming from the same batch.

A sample of frozen strawberries obtained from a case of the second wave in Berlin that had not eaten any other strawberry products in the incubation period tested negative for HAV. Samples of two frozen strawberry cakes from brand A obtained from two different cases also tested negative for HAV.

Food safety authorities have not reported product recalls from the German market in relation to this outbreak.

## Outbreak control measures

For 2 weeks after onset of symptoms, cases were excluded from school or childcare facilities and workplaces that involved handling and preparing food for others. Close contacts, i.e. household members and partners, of cases were offered post exposure prophylaxis using monovalent HAV vaccine and advised to strictly adhere to hand hygiene recommendations for a minimum of 2 weeks after post exposure prophylaxis.

A general recommendation for consumers to thoroughly heat products containing frozen berries was released on the website of the Federal Office of Consumer Protection and Food Safety (BVL) in late July 2019.

## Discussion

We report two hepatitis A outbreak waves in Germany attributable to the same HAV strain that caused previous outbreaks in Sweden and Austria linked to consumption of frozen strawberries. Epidemiological investigations in Germany provided strong evidence for frozen strawberry cake from brand A as the main vehicle in both German outbreak waves. Interviews with 46 cases showed that the majority (n = 34) could be explained by reported consumption of products containing frozen strawberries. CCS2 was conducted independently of CCS1, i.e. without foreknowledge of CCS1 results, and in both case–control studies, high ORs were obtained for frozen strawberry cake, particularly of brand A in CCS1. Furthermore, the major role of this product in the transmission of HAV in both outbreak waves was supported by comparing consumption data of cases with purchase data of a large consumer panel.

Despite the strong epidemiological evidence for frozen strawberry cake from brand A as the main vehicle, additional frozen strawberry products must be considered. Especially in the first wave, there were also cases who exclusively consumed other frozen strawberry products, which may suggest that those also played a role as vehicle(s).

Hepatitis A outbreaks linked to frozen berries have been frequently reported in recent years [14-16]. Distribution routes of frozen berries have international dimensions and large-scale, multistate outbreaks in the EU affecting hundreds of cases have occurred in the past [17]. Week-long stability of the hepatitis A virus particles in fresh produce stored at ambient temperatures [18], with stability being even longer in frozen food, combined with a lack of virus inactivating procedures, e.g. heat treatment of fruit, enables its dissemination.

The sequence identity of the outbreak strains, the timing of the first cases occurring in Germany, directly following the outbreaks in Sweden and Austria, and the similarity of cases’ exposures suggests these outbreaks are connected. Trace back investigations both in Sweden and Austria in 2018 were able to link the frozen strawberries to a common producer in Poland, and suggested that strawberries originating from Poland were the vehicle. Our phylogenetic analyses linked the isolated strain to similar strains formerly characterised from strawberries, sewage and stool in Egypt [11,12]. The outbreak subgenotype IB strain is therefore very likely similar to circulating strains in Egypt. This might indicate that production and/or contamination of strawberries with HAV could have occurred in Egypt.

Contamination of berries with enteric viruses can occur in various ways. The most likely route of contamination is contaminated water used for irrigation or processing of fruits. The occurrence of human enteric viruses, including HAV, was shown in irrigation water and fresh produce samples collected from urban agricultural farms in different regions in Egypt and may be a consequence of an irrigation process that uses contaminated surface water [19]. Interestingly, Polish producer Y, to whom implicated strawberries in the Swedish and Austrian outbreaks were traced back to, has received Egyptian strawberries from a German distributor. Frozen strawberries from the same batch were distributed to producer of strawberry cake brand A in Germany. Hence, a single contaminated batch from Egypt could have caused the entire outbreak.

The negative HAV NAT test result of the retention sample of the strawberries provided by the German distributor does not rule out contamination of the product. Detection of HAV in food samples, especially in berries, is extremely difficult and often unsuccessful [20,21]. Considering the low minimum infectious dose of HAV, the established test methods might not be sensitive enough to detect particularly low levels of viral load in contaminated food. Heterogeneous virus contamination and a generally low viral load in berries combined with a difficult sample matrix often leads to very low virus recovery rates, ranging from 0 to 1% [22]. Moreover, because of the long incubation period of HAV potentially implicated food items have most likely been eaten or disposed of at the time of case investigation and thus not available for analysis.

### Limitations

A substantial underestimation of outbreak cases has to be assumed because: (i) not all HAV infections are diagnosed and notified and (ii) only a small fraction of those identified, e.g. 17% of all notified cases fulfilling the case definition for surveillance in 2018, have isolates sent in for molecular sequencing. Additionally, because forwarding of diagnostic samples for sequencing of HAV is voluntary, the proportion of sequenced samples varies considerably by state. As with most CCS in investigations of food-borne outbreaks, our investigations are subject to incomplete and inaccurate recall, especially because of the long incubation period of HAV and time periods of 2 months before symptom onset were queried, often after additional delays. Furthermore, cases used in hypothesis generation were also used in hypothesis testing, i.e. in the CCS; the selection of controls in both studies was based on convenience samples, which may not be representative for the population that gave rise to the cases with respect to their (strawberry) consumption behaviour. Furthermore, the small number of cases in both CCS resulted in large confidence intervals of the obtained ORs. However, purchase data, which is devoid of these common biases, and which we used to additionally assess background purchase rates of frozen strawberry cake brand A strongly supports the results of the CCS.

Thus far, the outbreak strain could not be detected in frozen strawberries in Germany, though it must be taken into account that the detection of HAV in food samples, especially in berries, is particularly difficult. As demonstrated in previous outbreaks [23,24], where microbiological evidence was lacking or obtained late, epidemiological evidence is valuable not only to initiate and guide food investigations, but also to trigger preventive measures, i.e. recall of products. Therefore, food safety measures should not rely on microbiological evidence alone.

Many uncertainties remain regarding distribution routes, the mechanism of contamination, the role of other frozen strawberry products in this outbreak and, if one or multiple shipments were involved. The last sporadic case belonging to this outbreak was identified in February 2020 (disease onset January 2020), probably attributable to the long shelf lives of frozen vehicle(s).

## Conclusions

The recurrence of the outbreak strain underlines the importance of investigating food-borne outbreaks and complete trace back of affected products. The possibility of a timely recall of potentially contaminated products in international outbreaks caused by food with complex supply chains has to be ensured. Otherwise, resurgence of an outbreak can occur even months after its apparent abatement. This is especially relevant for frozen food products, which typically have a shelf life of 2 years or more. Frozen berries are a frequent vehicle for hepatitis A outbreaks and production conditions should be critically appraised to identify and eliminate potential sources of contamination. Evidence produced by epidemiological investigations, just like microbiological evidence, should suffice to initiate trace back and food investigations, particularly because epidemiological evidence is often available before microbiological evidence is present, if present at all. Molecular typing of human HAV isolates has again proven indispensable for the detection, investigation and monitoring of geographically dispersed or protracted outbreaks.
